# Synthesis and Evaluation of L-Glutamic acid Analogs as Potential Anticancer Agents

**DOI:** 10.4103/0250-474X.41467

**Published:** 2008

**Authors:** C. L. Vishwanathan, S. Deb, A. Jain, T. Lokhande, Aarti Juvekar

**Affiliations:** Department of Pharmaceutical Chemistry, Bombay College of Pharmacy, Kalina, Santacruz (E), Mumbai-400 098, India; 1 Department of Chemotherapy, Tata Memorial Centre, Advanced Center for Treatment, Research and Education in Cancer (ACTREC), Kharghar, New Mumbai-410 208, India

**Keywords:** L-glutamic amides, L-glutamic acid hydrazides, anticancer activity

## Abstract

Four N-(benzenesulfonyl)-L-glutamic acid bis(p-substituted phenylhydrazides) were synthesized and evaluated for anticancer activity *in vitro* in DU-145 and PC-3 prostate cancer and in COLO-205 colon cancer cell lines by MTT assay. The analog with the nitro group substitution exhibited potent activity (% Inhibition 84.7 and 72.0 in DU-145 and PC-3 respectively at 80 μg/ml concentration). Another series of substituted 1-(benzenesulfonyl)-5-oxopyrrolidine 2-carboxamides (11a-f) were synthesized and evaluated for anticancer activity *in vitro* in colon (COLO-205), breast (Zr-75-1) and prostate (PC-3) cancer cell lines by MTT assay using adriamycin as standard. Test compounds 11a-c showed potent activity (% Inhibition 61.2 to 79.2 at 20 μg/ml and 67.2 to 87.2 at 40 μg/ml) in PC-3 cell line which is superior to the activity of Adriamycin. In comparison compounds 11d-f were less potent. In Zr-75-1 cell line 11a-e showed % inhibition ranging from 32.4 to 54.9 at 10 μg/ml concentration while in COLO-205 cell line 11a-f showed poor activity.

L-Glutamic acid plays an important role in the biosynthesis of purine and pyrimidine bases of DNA and RNA^1^. It is metabolized to L-glutamine by L-glutamine synthetase and this metabolic process is essential for normal maintenance of cells. The synthesis of L-glutamine is hindered in neoplastic cells due to lower reactivity of L-glutamine synthetase. Thus antagonists of this enzyme can interfere with the metabolic role of L-glutamine and act as anti-cancer agents^2^. Azaserine and 6-diaza-5-oxo-L-norleucine antagonized the metabolic process involving L-glutamine and exhibited antitumor activity in animal models^3^. L-glutamic acid γ-(4-hydroxyanilide) a growth regulatory substance isolated from mushroom *Agaricus bisporous*was found to inhibit B16 mouse melanoma cells in culture^4^.

Similarly an arylamide derivative of L-threo-γ-hydroxy glutamic acid isolated from *Justica ghiesbreghtiana* was found to be active against various tumors^5^. The synthetic amides of L-glutamic acid also exhibited activity against Ehrlich ascites carcinoma^6^. In the present work it is proposed to synthesize novel N-(benzenesulfonyl)-L-glutamic acid bis(p-substituted phenylhydrazides) and substituted 1-(benzenesulfonyl)-5-oxopyrrolidine-2-carboxamides and evaluate them in cancer cell lines to ascertain their anticancer activity.

All melting points were recorded on Thermonik melting point apparatus and are uncorrected; Fourier-Transform Infrared (FT-IR) spectra (KBr discs) were recorded in Jasco FT-IR 5300 instrument. ^1^H-NMR spectra were recorded in Varian spectrophotometer at 60 MHz for first set of compounds and at 300 MHz for second set of compounds. The chemical shift values are reported in δ units (ppm) relative to internal standard tetramethylsilane (TMS). Elemental analysis values for final compounds were within ±0.4 % of theoretical value.

A group of four N-(benzenesulfonyl)-L-glutamic acid bis(p-substituted phenylhydrazides) were synthesized by condensing L-glutamic acid with benzenesulfonyl chloride in presence of aqueous NaOH at 70^º^followed by acidification with aqueous HCl and extraction in dry ethyl acetate. The sulfonamide formed was converted to acid chloride using thionyl chloride and then condensed with excess of p-substituted phenylhydrazines to get bisamides (1-4). The physical constant and spectral characteristics are given in Table 1.

**TABLE 1:** PHYSICAL AND SPECTRAL DATA OF N-(BENZENESULFONYL)-L-GLUTAMIC ACID BIS (P-SUBSTITUTED PHENYLHYDRAZIDES) (1-4) 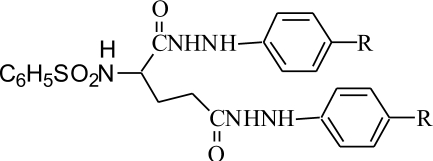

Compound	R	M.P.^º^	Yield %	IR (KBr cm^-1^)	^1^H-NMR (DMSO-D_6_) δ ppm
1	H	190	65	3341, 3221 (N-H stretch), 1684 (C=O stretch), 1358, 1168 (S=O stretch).	1.7-2.7 (5H, m, -CH CH_2_ CH_2_), 4.3 (2H, s, 2 Ar-NH-), 6.9-8.0 (15 H, m, Ar-H), 9.3 (1H, s, -SO_2_NH, 10.1 (2H,s, 2 HN-CO)
2	NO_2_	202	67	3200 (N-H stretch), 1672 (C=O stretch), 1342, 1172 (S=O stretch).	1.8-3.5 (5H, m, -CH CH_2_ CH_2_), 4.4 (2H,s, 2 Ar-NH-), 6.92-8.30 (13 H, m, Ar-H), 9.5 (1H, -SO_2_NH, 10.1 (2H, s, 2 HN-CO)
3	Cl	113-114	41	3312 (N-H stretch), 1703 (C=O stretch), 1356, 1170 (S=O stretch).	2.07-3.5 (5H, m, -CHCH_2_CH_2_), 4.2 (2H, s, 2 Ar-NH-), 6.6-7.93 (13 H, m, Ar-H), 8.9 (1H, s, -SO_2_NH, 10.2 (2H, s, 2 HN-CO)
4	CH_3_	212-214	57	3225, 3209 (N-H stretch), 1684 (C=O stretch), 1361, 1168 (S=O stretch).	2.35 (6H, s, 2CH_3_), 2.50-3.56 (5H, -CHCH_2_CH_2_), 4.0 (2H, s, 2 Ar-NH-), 6.54-7.93 (13H, m, Ar-H), 9.5 (1H, -SO_2_NH, 10.0 (2H, s, 2 HN-CO)

General procedure used for the synthesis of N-substituted-5-oxopyrrolidine-2-carboxylic acid is as follows, in a 250 ml round bottom flask fitted with a CaCl_2_ guard tube, 20 g (0.066 mol) of N-p-toluenesulfonyl-L-glutamic acid (7) was taken and 25 ml (0.264 mol) of freshly distilled POCl_3_was added to it gradually with cooling of the flask in ice bath. Then 2-3 drops of DMF was added and the reaction mixture stirred for 3-4 h at room temperature. The reaction mixture was poured with stirring into crushed ice and the precipitate formed was extracted in chloroform. Chloroform extract dried over anhydrous MgSO_4_. Removal of solvent gave 8 in pure form. Compound 8 (R=CH_3_), Yield: 95 %; Mp 178-180°. IR (KBr, cm^-1^): 3414 (O-H stretch), 2932 (C-H stretch), 1736 (C=O stretch, lactam), 1707 (C=O stretch, acid), 1356 (S=O stretch). ^1^H NMR (CDCl_3_) δ ppm: 1.9 (2H, m, -CH_2_), 2.1 (3H, s, -CH_3_), 2.2 (2H, t, -CH_2_ -C=O), 4.9 (1H, t, NC-H), 7.3 (2H, d, Ar-H), 8.0 (2H, Ar-H), 9.0 (1H, s, -OH).

The above formed acid was converted to acid chloride by heating for an hour with 15 ml of SOCl_2_and with addition of 2 drops of DMF and removing the excess of SOCl_2_by vacuum distillation. Compound 9 (R=CH_3_), Yield: 95 %; Mp 71-72°. Synthesis of substituted 1-(benzenesulfonyl)-5-oxopyrrolidine 2-carboxamides (11a-f) was achieved by adding gradually to a solution of 3.9 g (0.012 mol) of 9 (R=CH_3_) in 5 ml of chloroform 1.46 g (0.020 mol) t-butylamine (10b) dissolved in 5 ml of chloroform with temperature maintained at 0°. The temperature was then raised to 50° and the mixture heated for 5-6 h. The chloroform solution was washed several times with water to remove unreacted amine, dried and then concentrated in vacuum. The amide obtained was purified by recrystallization with aqueous methanol or with ethyl acetate-hexane mixture (4:1). Compound 11b: Yield: 78 %; Mp 202-203°. IR (KBr, cm^-1^): 3290 (N-H stretch), 2966 (C-H stretch); 1751 (C=O stretch, lactam), 1651 (C=O amide). ^1^H NMR (CDCl_3_) δ ppm: 1.0 (3H, t, -CH_3_), 1.2 (2H, m, -CH_2_), 1.4 (2H, q, -CH_2_), 1.8 (2H, m, -CH_2_), 2.2 (2H, t, H_2_ C-CO), 2.4 (2H, t, N-CH_2_), 2.5 (3H, s, Ar-CH_3_), 5.3 (1H, m, -N-C-H), 7.3 (2H, d, Ar-H), 8.0 (2H, dd, Ar-H).

Test compounds 1-4 were evaluated for cytotoxicity in DU-145, PC-3 (prostate) and colo-205 (colon) cancer cell lines, while test compounds 11a-f were tested in PC-3, colo-205 and Zr-75-1 (breast) human cancer cell lines. All compounds were evaluated *in vitro* at 10, 20, 40 and 80 mg/ml concentrations using Adriamycin as standard and using MTT assay^7^.

Cell suspension 90 μl /well (5×10^3^cells) was added to 96 wells and incubated at 37° for 24 h in 5% CO_2_incubator. Ten microlitres of either solutions of test compounds or solution of Adriamycin were added to the wells and the plates were incubated at 37° for 48 h in 5 % CO_2_ incubator. MTT reagent 10 μl (0.5%) was then added to the wells and further incubated for 6 h. The supernatant was then carefully removed and the formazan crystals formed were dissolved in 100 μl of acidified isopropanol (0.04 N HCl in isopropanol) and the absorbance of the solution was then read on spectrophotometer at a wavelength of 540 nm. The absorbance values were used to calculate % inhibition at various concentrations. The results are average values of three experiments.

A series of substituted 1-(benzenesulfonyl)-5-oxopyrrolidine-2-carboxamides (11a-f) were synthesized by cyclizing compound 7 using PCl_5_ and reacting with amines (10a-f). This method gave colored impurities which were difficult to remove. The above reaction when carried out with excess POCl_3_, instead of PCl_5_, generated less colored impurities but complete removal of excess POCl _3_ at the end of the reaction was difficult. Hence in the reaction with POCl_3_after cyclisation of 7, the reaction mixture was poured into crushed ice to destroy excess POCl_3_and the product isolated was the free acid (8). The free acid was then converted to the acid chloride using SOCl_2_ (Scheme 1). The structure & physical constants & yields of the final steps are given in Table 2.

**Scheme 1:**
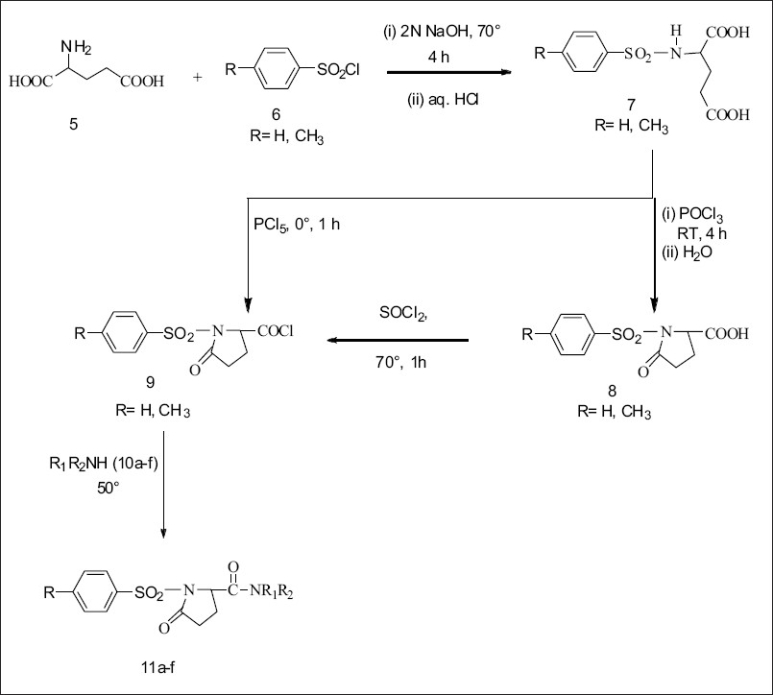
Synthesis of substituted 1-(benzylsulfonyl)-5-oxopyrrolidine-2-carboxamides. 11a: R -CH_3_, R_1_ -H, R_2_ n-C_3_H_7_; 11b: R -CH_3_, R_1_ -H, R_2_ n-C_4_H_9_; 11c: R -CH_3_, R_1_ -H, R_2_ -NH_2_; 11d: R -CH_3_, R_1_ -C_2_H_5_, R_2_ -C_2_H_5_; 11e: R -H, R_1_ -H, R_2_ n-C_3_H_7_; 11f: R -H, R_1_ -H, R_2_ n-C_4_H_9_.

**TABLE 2:** PHYSICAL AND SPECTRAL DATA OF 1-(BENZENESULFONYL)-5-OXOPYRROLIDINE-2-CARBOXAMIDES (11a-f) 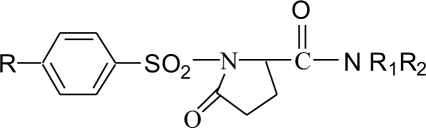

Compound	R	R1	R2	Yield %	M.P.º	IR (KBr cm^-1^)
11a	CH_3_	H	n-C_3_H_7_	76	194-195	3300 (N-H stretch), 1751 (C=O stretch, lactam), 1653(C=O stretch), 1359(S=O stretch).
11b	CH_3_	H	n-C_4_H_9_	78	202-203	3290 (N-H stretch), 1751 (C=O stretch, lactam), 1651 (C=O stretch), 1356 (S=O stretch).
11c	CH_3_	H	-NH_2_	91	178-179	3321(N-H stretch), 1732 (C=O stretch, lactam), 1648 (C=O stretch), 1350 (S=O stretch).
11d	CH_3_	C_2_H_5_	C_2_H_5_	89	143-145	3375(N-H stretch), 1738 (C=O stretch, lactam), 1660 (C=O stretch), 1358 (S=O stretch)
11e	H	H	n-C_3_H_7_	82	205-208	3362(N-H stretch), 1732 (C=O stretch, lactam), 1664 (C=O stretch), 1348 (S=O stretch).
11f	H	H	n-C_4_H_9_	79	214-215	3369(N-H stretch), 1718 (C=O, stretch lactam), 1687 (C=O stretch), 1356 (S=O stretch).

Test compound 2 was found to be potent with % inhibition of 84.7, 86.6 and 42.2 in DU-145, PC-3 colo-205 cancer cell lines respectively at 80 μg/ml concentration, but was less potent at lower concentrations. Compounds 1, 3 and 4 exhibited poor activity at all doses studied (Table 3).

**TABLE 3:** IN VITRO ANTICANCER ACTIVITY OF TEST COMPOUNDS (1-4) ON DU-145, PC-3 AND COLO-205 CELL LINES

Compound	% Inhibition at 80 μg/ml in		
	DU-145	PC-3	COLO-205
1	1.3	0.0	7.9
2	84.7	72.0	42.2
3	0.5	0.0	10.7
4	0.0	0.0	28.1
Adriamycin	86.6	98.3	97.8

*In vitro* anticancer activity of test compounds (1-4) using MTT colourimetric assay in DU-145, PC-3 (prostate) and COLO-205 (colon) human cancer cell lines

Test compounds 11a-f exhibited significant inhibitory activity in PC-3 cell line and 11a-c were more active than Adriamycin at 10, 20 and 40 μg/ml concentrations. In Zr-75-1 cell line the % inhibition ranged from 31.5 to 54.9 at 10 μg/ml concentrations but activity dropped with increasing concentrations. In colo-205 cell line, test compounds 11a-f as well as Adriamycin showed reduced activity at all concentrations. The percentage inhibition determined is reported in Table 4. The present work led to synthesis of two series of L-glutamic acid amides and among these, tests compounds 2 and 11a-c exhibited potent anticancer activity in some of the cell lines tested.

**TABLE 4:** *IN VITRO* ANTICANCER ACTIVITY OF TEST COMPOUNDS (11a-f) IN PC-3, COLO-205 AND ZR-75-1 CANCER CELL LINES

Compound	% Inhibition at various concentrations in μg/ml in											
	
	PC-3				Zr-75-1				COLO-205			
	10	20	40	80	10	20	40	80	10	20	40	80
11a	52.1	70.7	81.1	45.5	32.4	0.0	0.5	0.0	1.3	2.0	4.9	1.5
11b	63.6	79.2	67.2	55.9	31.5	11.8	5.6	5.9	2.1	3.2	4.6	2.3
11c	59.1	61.2	82.9	45.9	53.8	5.8	2.3	3.4	3.9	3.8	3.0	1.2
11d	27.9	40.9	45.6	23.3	51.6	10.0	2.7	6.1	2.2	1.6	0.8	0.9
11e	31.9	65.2	19.3	05.3	54.9	12.9	6.4	5.3	0.4	1.1	0.6	0.7
11f	19.2	0.73	0.0	0.0	0.0	0.0	0.0	0.0	0.9	11.4	3.7	5.2
Adriamycin	56.0	59.2	61.2	97.2	60.9	78.1	83.8	87.8	50.7	52.6	54.9	57.6

*In vitro* anticancer activity of test compounds (11a-f) using MTT colourimetric assay in PC-3 (prostate), COLO-205 (colon) and ZR-75-1 (breast) human cancer cell lines
